# Estimating Genome-Wide Significance for Whole-Genome Sequencing Studies

**DOI:** 10.1002/gepi.21797

**Published:** 2014-02-14

**Authors:** ChangJiang Xu, Ioanna Tachmazidou, Klaudia Walter, Antonio Ciampi, Eleftheria Zeggini, Celia M T Greenwood, and the UK10K Consortium

**Affiliations:** 1 Department of Epidemiology, Biostatistics and Occupational Health, McGill UniversityMontreal, Canada; 2 Lady Davis Institute for Medical Research, Jewish General HospitalMontreal, Canada; 3 Department of Human Genetics, Wellcome Trust Sanger InstituteHinxton, United Kingdom; 4 Department of Oncology, McGill UniversityMontreal, Canada; 5 Department of Human Genetics, McGill UniversityMontreal, Canada

**Keywords:** genome-wide significance, rare-variant analysis, multiple testing, sliding windows, whole-genome sequencing, region-based tests, effective number of independent tests

## Abstract

Although a standard genome-wide significance level has been accepted for the testing of association between common genetic variants and disease, the era of whole-genome sequencing (WGS) requires a new threshold. The allele frequency spectrum of sequence-identified variants is very different from common variants, and the identified rare genetic variation is usually jointly analyzed in a series of genomic windows or regions. In nearby or overlapping windows, these test statistics will be correlated, and the degree of correlation is likely to depend on the choice of window size, overlap, and the test statistic. Furthermore, multiple analyses may be performed using different windows or test statistics. Here we propose an empirical approach for estimating genome-wide significance thresholds for data arising from WGS studies, and we demonstrate that the empirical threshold can be efficiently estimated by extrapolating from calculations performed on a small genomic region. Because analysis of WGS may need to be repeated with different choices of test statistics or windows, this prediction approach makes it computationally feasible to estimate genome-wide significance thresholds for different analysis choices. Based on UK10K whole-genome sequence data, we derive genome-wide significance thresholds ranging between 2.5 × 10^−8^ and 8 × 10^−8^ for our analytic choices in window-based testing, and thresholds of 0.6 × 10^−8^–1.5 × 10^−8^ for a combined analytic strategy of testing common variants using single-SNP tests together with rare variants analyzed with our sliding-window test strategy.

## Introduction

Complex-trait genetic association studies aim to identify robust associations between genotype and phenotype in order to enhance our understanding of the underlying biological processes contributing to the trait of interest. The field of complex-trait genetics has thus far focused on the study of common (minor allele frequency [MAF] ≥ 0.05) variants through candidate gene studies and, in recent years, through genome-wide association scans (GWAS). The candidate gene study era was unsuccessful in identifying many reproducible associations, partly due to the liberal thresholds used to declare statistical significance, and the issue of multiple testing became even more pronounced with the advent of GWAS. To guard against lenient significance thresholds, a genome-wide significance level was estimated, based on a Bonferroni correction for the number of independent (i.e., uncorrelated) common variants across the genome: 5×10^−8^ [Dudbridge and Gusnanto, 2008; Gao et al., 2008; Pe'er et al., 2008]. Adherence to this threshold has served the field well, with a very high proportion of genome-wide significant signals withstanding replication.

Common-frequency variants explain only a small proportion of complex-trait heritability. Following advances in large-scale genotyping and next-generation sequencing technologies, low-frequency (0.01 < MAF < 0.05) and rare (MAF < 0.01) variants are increasingly becoming the the focus of genetic association studies, as they are hypothesized to have larger effect sizes, more readily interpretable functions and possible translational potential. Whole-exome sequencing (WES) and whole-genome sequencing (WGS) studies identify almost all sequence variation in the targeted genomic regions, and the variants identified are often extremely rare or unique; hence, the number of variants observed increases with sample size.

Analysis of WGS and WES studies usually includes not only single-variant tests, but also region-based tests, which aggregate information across multiple rare variants in order to boost power lost to the combination of low frequency and modest sample size. Numerous region-based tests for rare genetic variation have been proposed. These rely on the initial definition of an interesting region or “functional unit,” which is intuitive for WES but can be defined in various different ways for WGS (e.g., genes only, regulatory regions, sliding windows with or without overlap). Calculations to estimate the significance thresholds then need to be based not only on the correlations between single-point (variant-specific) tests but also on the dependence between window-based tests. Although rare variants are known to display lower levels of linkage disequilibrium than common variants, for test statistics applied to regions or windows, especially partially overlapping windows, the dependence structure is not well understood.

Therefore, in association studies of sequencing data, the number and frequency of variants tested are very different from the GWAS era, and the choice of potential test statistics is large. Therefore, it is currently unclear what the respective WES and WGS genome-wide significance thresholds should be. Although a simple and commonly used significance threshold for WES is simply (0.05/#genes), it is likely that the thresholds which accurately control the type 1 error should be study-specific, taking into account the analysis choices as well as key study design parameters including the size of the sequenced regions and the sample size, in order to identify the total number of independent tests carried out.

We define genome-wide significance using the definition of family-wise error; our goal is to control the probability of making one or more false discoveries at level α. The family-wise error rate, α, can be capped by testing at a significance level, *α_c_*, which has been adjusted for the number of independent tests performed, such that *α_c_* = 1 − (1 − *α*)^(1/*me*)^ ≈ *α*/*m_e_*, where *m_e_* is the number of independent tests performed, which will be smaller than the actual number of tests, *m*, due to dependence between test statistics. In particular, for window-based analysis of WGS data, we would like to understand the magnitude of the correlations between nearby test statistics and the genomic distance over which correlations extend; this has not been previously explored in detail.

We use two approaches for understanding the dependence structure and for estimating the effective number of independent tests, one based on correlations and one using simulations. Association analysis of sequencing studies usually includes both single-variant tests at markers with MAF large enough to enable good power, as well as window-based tests for rare genetic variation. We therefore study dependence patterns for the combination of both types of tests. The resulting understanding will allow us to estimate the effective number of independent tests, *m_e_*, for a complete analytic strategy, after estimating and adjusting for the correlations.

It is unlikely that window-based tests located on separate chromosomes or far apart on the same chromosome will display much correlation. Therefore, here we study the behavior of the effective number of independent tests as a function of the size of a genomic region under analysis. Notably, we propose a strategy of predicting or extrapolating from results obtained on one subset of the genome to genome-wide analysis, in order to make the computations efficient and feasible. Finally, we recommend reasonable significance thresholds for a plausible WGS study analytic plan employing both single-point analysis of common variants and a specific sliding-window implementation of region-based tests of rare variants, for the detection of complex-trait associations.

## Methods

### Dataset

Performance and results are illustrated with an interim release of sequencing data from the UK10K project (http://www.uk10k.org), where almost 10,000 individuals are undergoing WGS. The UK10K project is the largest WGS study to date generating data one order of magnitude deeper compared to the 1000 Genomes Project. Sequencing data on chromosome 3 were obtained, consisting of 2,577,674 genetic variants in 2,432 individuals. There were 23,096 sites where no minor alleles were observed after data cleaning; these variants were excluded. Rare genetic variants were defined using three thresholds MAF <0.005, MAF <0.01, and MAF <0.05 leading to 1,779,499 variants for MAF <0.005, 1,853,923 variants for MAF <0.01, and 2,046,466 for MAF <0.05.

Windows for analysis were defined to contain 50 rare variants, where rare is defined using MAF less than 0.005, 0.01, or 0.05, and adjacent windows overlapped by 25 variants. This choice was derived from earlier WES analysis of UK10K data where a maximum of 50 variants were allowed in a single window for analysis. Because the genome-wide window definitions are independent of genomic annotations, tiling windows that overlap by half were chosen in order to include the same variants in two different sets. The windows, therefore, vary in genomic length depending on the number of sequence variants identified and the MAF threshold used to define rarity; there were 71,179 windows for MAF <0.005, 74,156 windows for MAF <0.01, and 81,858 for MAF <0.05 along chromosome 3.

### Test Statistics

For the genetic variants on chromosome 3 only, all SNPs with MAF greater than each chosen threshold were analyzed individually, and window-based tests were used to analyze variants with MAF less than the threshold. Windows were analyzed with SKAT [Wu et al., 2011], a simple burden test [Price et al., 2010], and SKAT-O [Lee et al., 2012]. For the burden test, we fit linear regressions between the phenotype and the total count of minor alleles across all variants in the window with MAF less than the chosen threshold. The popular SKAT test is sensitive to increased variance of the phenotype associated with the presence of rare genetic variants, and SKAT-O is a combined test that finds the optimal combination of the SKAT statistic and a burden test. These tests represent several of the most commonly used window-based tests, and have demonstrated good power across a range of allelic architectures. Single-marker tests were performed with linear regression on the minor allele count, leading to a *F*_1,*N*−2_ test, for a sample size of *N*.

### Expected Correlations Under the Null Hypothesis

The expected correlation between pairs of SKAT [Wu et al., 2011] statistics (*T_i_, T_j_*) can be analytically derived for a given set of genotypes, under the null hypothesis. The SKAT test statistic is


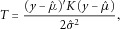


where *y* is a vector of phenotypes, 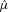 is the predicted mean of *y* under null hypothesis, *K* = (*GW*) (*GW*)' is the SKAT kernel matrix, which depends on the genotype matrix *G*, assumed to be centered, and a choice of variant weights *W*. Let *T_i_* and *T_j_* be two SKAT test statistics. Let 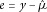 and *Q_i_* = *e*'*K_i_e*, where *K_i_* = (*G_i_ W_i_*) (*G_i_ W_i_*)'. Under the null hypothesis, *e* ∼ *N*(0, *σ*^2^*I*) and 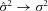. Hence, the correlations under the null can be calculated as


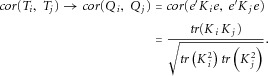


Expected correlations between burden tests can also be written analytically. For the genotype matrix *G* within a window, let *x* be the count of the number of minor alleles at rare variants in the window (or the row sum of *G*). For a simple linear regression test, the burden test statistic is given by


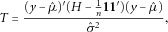


where *n* is the sample size, *H* is the hat matrix of the regression test, and **1** is a vector of ones. Let *T_i_* and *T_j_* be two burden test statistics, and let 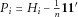. The correlations under the null can be calculated as


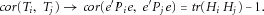


Obtaining an explicit expression for correlation between the SKAT-O statistics is not straightforward. Therefore, we have calculated expected correlations between window-based test statistics for burden and SKAT tests only. These calculations were performed for the genotype data on chromosome 3 from UK10K, using the sets of windows defined for rare variant thresholds of MAF = 0.01 and MAF = 0.05.

### Estimators of the Effective Number of Independent Tests, Based on the Eigenvalues From a Singular Value Decomposition of the Matrix of Correlations Between Test Statistics

Several different approaches have been proposed for estimating the effective number of independent tests given a correlation matrix [Chen and Liu, 2011; Cheverud, 2001; Dudbridge and Gusnanto, 2008; Gao et al., 2008; Li and Ji, 2005; Li et al., 2012; Moskvina and Schmidt, 2008; Patterson et al., 2006]. Some estimators are based on the eigenvalues of the correlation matrix, and others can be derived directly from the correlations without the need to calculate the eigenvalues [Chen and Liu, 2011; Cheverud, 2001; Moskvina and Schmidt, 2008]. Most of these methods have been used for single-SNP tests; we explored their performance using the calculated correlations between window-based burden and SKAT tests. We found particularly good performance from two of the eigenvalue-based methods, where results agreed well with those from simulations (described below) [Li and Ji, 2005; Li et al., 2012].

Let *m* be the number of tests performed in a chosen genomic region or section, and let *λ_i_*, *i* = 1, … *m* be the eigenvalues of the matrix of correlations for all tests in this region. The two methods we have used for calculating the effective number of independent tests, *m_e_*, are the method of Li and Ji [2005]


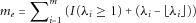


and the method of Li et al. [2012], which is known to give estimates slightly smaller than Li and Ji [2005],


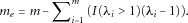


### Simulation Design

Calculation of correlations and eigenvalues becomes computationally infeasible for large numbers of tests. Hence, we also undertook a complementary approach based on simulation of phenotypes, also using the WGS data from chromosome 3 in the UK10K project. One thousand sets of normally distributed phenotypes were generated (under the null hypothesis of no association), and both single-marker (linear regression) and window-based tests (SKAT, burden, and SKAT-O) were performed across the chromosome 3 data for each set of phenotypes.

### Calculating the Predicted Effective Number of Independent Tests From Simulated Data, and Predicting the Significance Threshold Genome Wide by Extrapolation

For each simulated phenotype, we calculated and stored all single-SNP test *P*-values and all window test *P*-values for MAF thresholds of 0.005, 0.01, and 0.05. We then partitioned the chromosome into a series of sections of equal size, ranging from 1,024 equally sized sections up to the entire length of chromosome 3. For example, for a MAF threshold of 0.01, the smallest sections contained *m* = 72 or 73 window tests each (74,156/1,024). For each section, we calculated the minimum *P*-value for the window tests, for the single-SNP tests, and for both windows and single SNPs combined, for each of the 1,000 simulations. The fifth percentile of the minimum *P*-values was identified within each section, and then the −log_10_(*P*) values for this percentile were averaged across all sections containing the same number of tests. These numbers are then empirical estimates of the (−log of the) necessary significance thresholds for FWER = 5%, for genomic pieces of varying sizes corresponding to the different sections; equivalently, these are empirical estimates of 0.05/*m_e_* (after antilog transformation), where *m_e_* is the effective number of independent tests.

We then fit linear regressions predicting the empirical thresholds as a function of −log_10_(0.05/*m*), for *m* tests. To estimate the number of tests that would be performed genome-wide, we assumed that the density of variants is approximately equally distributed across all chromosomes. Because the whole genome is approximately 15.6 times longer than chromosome 3, we therefore created a new point on the *x*-axis at x* = −log_10_(0.05/(15.6*m_max_*_3_)), where *m_max_*_3_ is the total number of tests on chromosome 3 (e.g., *m_max_*_3_ = 74,156 for window tests and MAF threshold = 0.01). Let the predicted value from the linear regression be y* = *b*_0_ + *b*_1_x*; hence, the predicted significance threshold is therefore *α_c_* = 10^−(y*)^ and the effective number of independent tests genome-wide is predicted to be 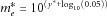.

## Results

### Results Based on Correlations

**Figure 1:**
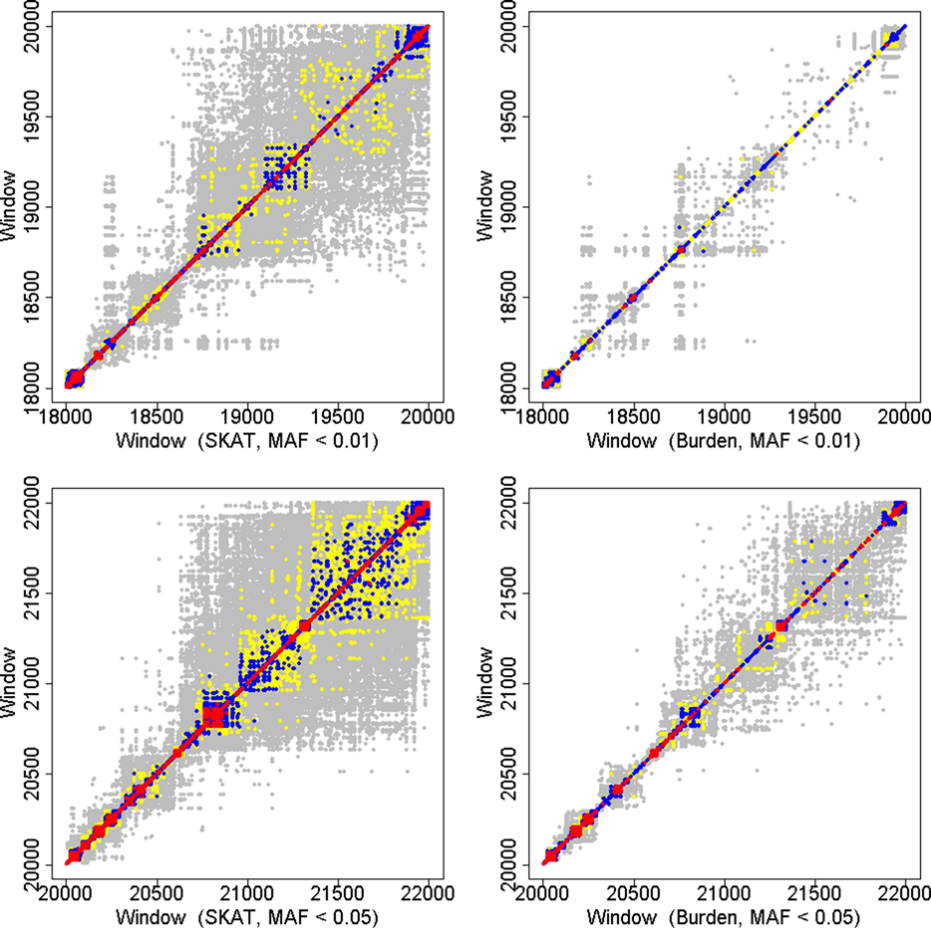
Correlations between 2,000 adjacent window-based tests statistics for SKAT and burden tests and for two different MAF thresholds. Because windows were defined to contain the same number of rare variants, the boundaries of the windows vary with MAF. Regions have been aligned so that the genomic region captured in the bottom row is contained within the genomic region at the top. The axes are the window numbers, counted from the 5' end of chromosome 3. Left column: SKAT tests. Right column: burden tests. Top row: MAF threshold = 0.01; bottom row: MAF threshold = 0.05. Gray: correlation > 0.1; yellow: correlation > 0.35; blue: correlation > 0.5; red: correlation > 0.75.

Figure1 displays heat maps of the genotype-based correlations calculated for a section of chromosome 3 containing 2,000 windows or 50,000 rare variants. Two different MAF thresholds are shown, for both burden and SKAT statistics. It can be seen that although correlations of 0.1 or more often extend across almost 1,000 windows, there are few substantial correlations that extend further than this. Also, correlations between windows are demonstrably more evident for SKAT tests than for burden tests. When comparing the two MAF thresholds, it can also be seen that the correlations are larger for the MAF threshold of 0.05 vs. 0.01. Because our windows were defined to contain 50 rare variants, each different MAF threshold induces a different set of window definitions. The two rows of Figure1 show an overlapping genomic region—the genomic region covered by the panels for MAF <0.05 is contained within the genomic region for MAF <0.01. Supplementary Figure S1A–C shows similar results for three additional regions of chromosome 3.

**Figure 2:**
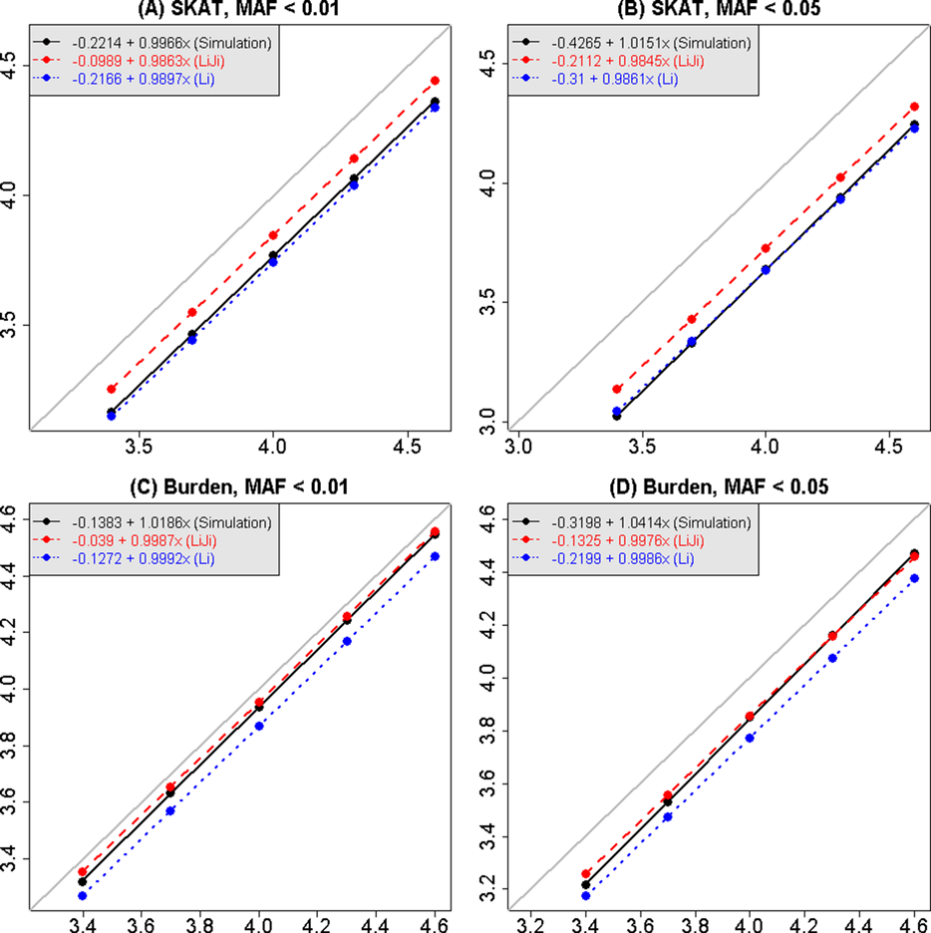
Estimates of significance thresholds as a function of the number of window tests, comparing estimates derived from the correlation matrices (the methods of Li et al., and Li and Ji) with estimates from simulations. Results are shown for MAF threshold = 0.05 and 0.01 and for SKAT and burden tests. The horizontal axis is −log_10_(0.05/*m*), for *m* tests; the maximum value corresponds to −log_10_(0.05/2,000), because the largest matrices we used were 2,000 × 2,000. Dots are the means of the estimated values of −log_10_(0.05/*m_e_*) across all sections of chromosome 3 of the same size, and linear regressions have been fit to each series points. The gray line is the line of equality, *y* = *x*.

In Figure2, multiple testing corrected significance thresholds, estimated by several different methods, are displayed for genomic regions of varying sizes up to 2,000 adjacent windows. Each panel shows mean values of −log_10_(0.05/*m_e_*) plotted against the Bonferroni equivalent (using *m* instead of *m_e_*). The means are averages of the estimated values for all genomic sections of the same size. For each method of estimation, the estimated values of *m_e_* increase linearly with the number of tests performed, *m*, and furthermore all estimates are below the diagonal line, implying that the effective number of independent tests is smaller than the total number of tests performed, as expected.

Examining the estimates derived from expected correlations between SKAT and burden tests, it can be seen that the stronger dependence between SKAT statistics (as was seen in Fig.1) leads here to less stringent significance thresholds for SKAT-based analysis, compared to burden tests. When comparing MAF thresholds, the dependence is stronger for MAF < 0.05, leading also to less severe penalties for multiple testing. Furthermore, it can be seen that the different methods for estimating *m_e_* lead to slightly different estimates; the method of Li et al. [2012] is more conservative than the result of Li and Ji [2005].

### Results Based on Simulations

The utility of the correlation-based estimates of *m_e_* is somewhat limited by the size of the matrix for which it is feasible to calculate the eigenvalues or store the full matrix of correlations; computations became difficult for genomic regions larger than approximately 2,000 windows. In addition, theoretical correlations were not available for SKAT-O, and furthermore, the expected correlations may be somewhat different from the true correlations, which depend on phenotype data. Therefore, we undertook a complementary approach, also using the WGS data from chromosome 3 in the UK10K project, where phenotypes were simulated under the null hypothesis and both single-marker and window-based tests were performed across the chromosome. This enabled us to empirically study the behavior of the minimum *P*-value for genomic sections of larger size.

**Figure 3:**
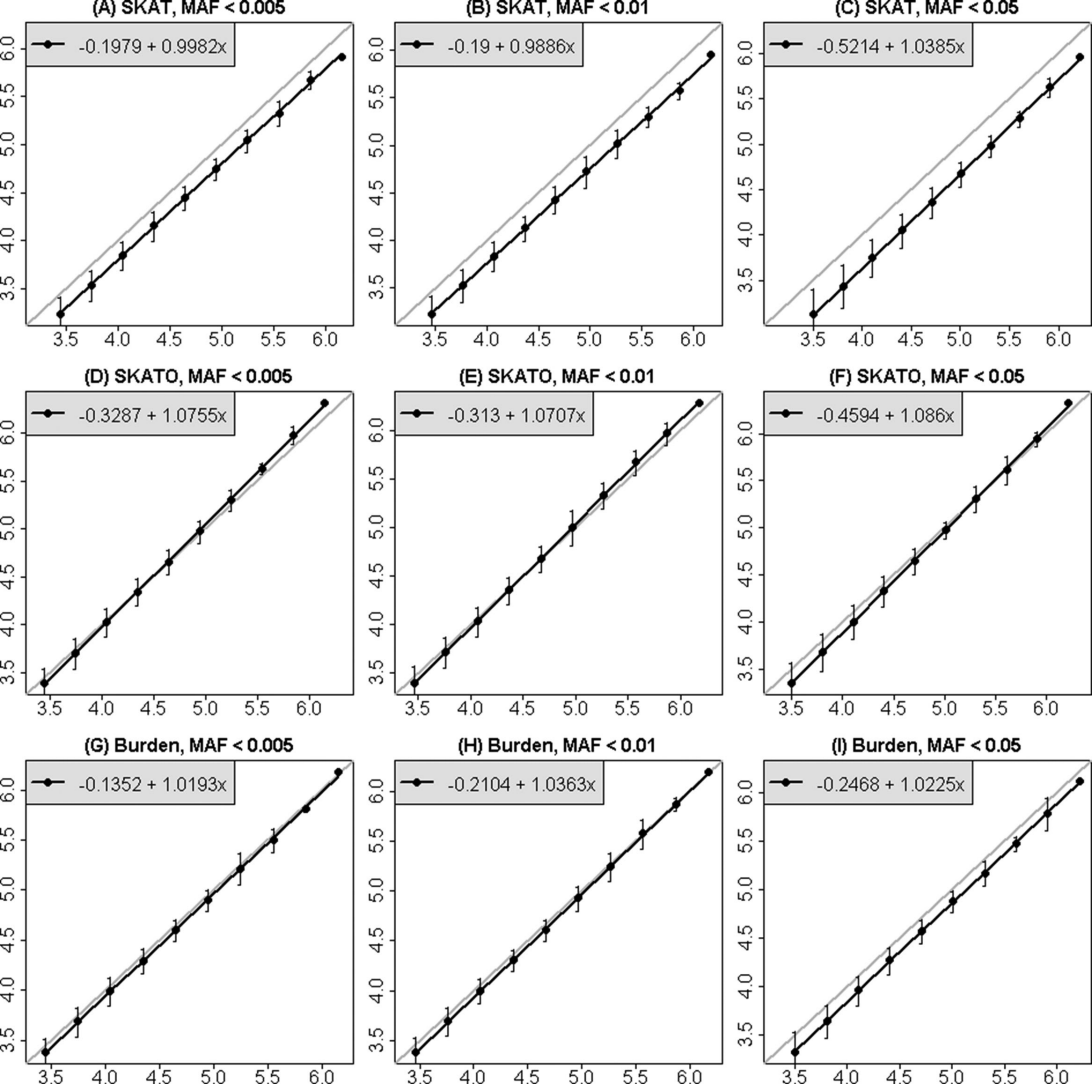
Estimates of genome-wide significance thresholds for window-based tests of rare variants, derived from simulations, for three MAF thresholds and three test statistics. The horizontal axis is −log_10_(0.05/*m*), for *m* tests on chromosome 3. Each point is the mean of −log_10_ of the estimated FWER at 5% for disjoint sections of chromosome 3 of the same size, and ±1.96*(SD) at each point. A linear regression was fitted to the points in each panel, and the gray line is the line of equality, *y* = *x*.

In Figure3, the estimated significance thresholds for window-based tests derived from simulations are displayed for three different MAF thresholds, and for three different test statistics for genomic sections increasing in length up to the entire length of chromosome 3. As in Figure2, it is clear that there is a linear relationship between the average estimated significance threshold and the Bonferroni threshold (on the −log_10_ scale) as the size of the genomic region analyzed increases. However, for a genomic region of a chosen size, there can be quite a lot of variability in the estimated significance thresholds, especially for smaller sizes. Figure3 also shows that significance thresholds for burden and SKAT-O statistics are closer to the Bonferroni correction than the SKAT statistic, and hence that these statistics are less correlated, confirming the result seen visually in Figure1 for the burden test. The effect of the MAF threshold on the conclusion is smaller, but for the SKAT statistic, it can be seen that higher MAF thresholds correspond to more interwindow correlation and hence to larger (less stringent) significance thresholds. This makes sense because there should be more linkage disequilibrium between the variants when a higher MAF threshold is used.

**Figure 4:**
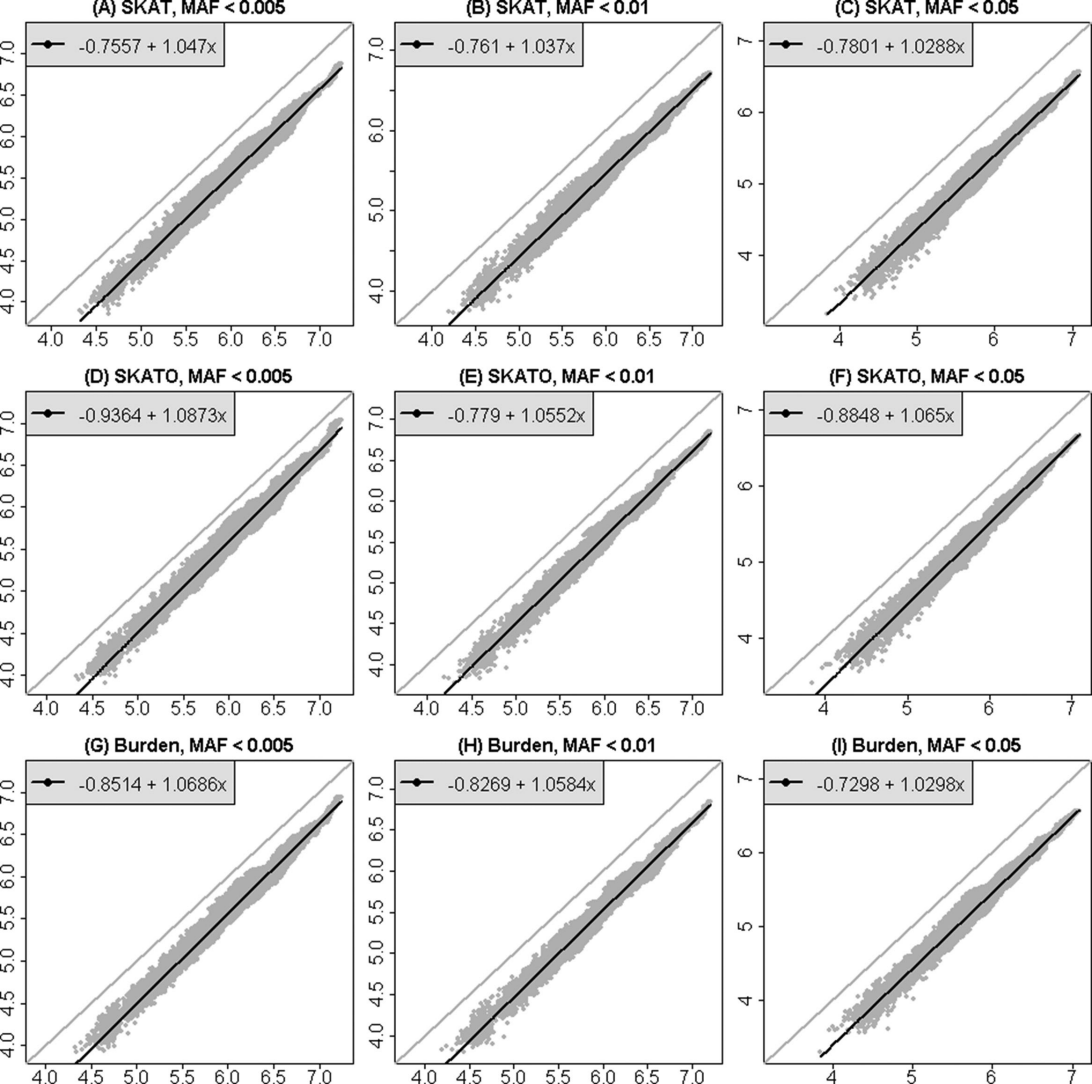
Estimates of genome-wide significance thresholds for a combined strategy including window-based tests of rare variants and single-marker tests of common variants. Results are derived from simulations, for three MAF thresholds and three test statistics. The horizontal axis is −log_10_(0.05/*m*), for *m* tests. Each dot is a single estimated value for −log_10_ of the FWER at 5% for sections of chromosome 3 of varying size. A linear regression was fit through all the data. The gray line is the line of equality, *y* = *x*.

The simulation approach also enables us to study the significance thresholds for a combination of window-based tests of rare genetic variation and single-marker tests of common variation. In Figure4, the necessary significance thresholds for controlling FWER at 5% are shown for genomic sections of varying size for this combined strategy. The variability across chromosomal sections of the same size is shown, as well as the linear relationship. All estimates here are well below the line of equality, *y* = *x*, demonstrating the well-known effect of linkage disequilibrium between common variants.

### Estimating Genome-Wide Significance

We have used the apparent linear relationships in Figures4 to infer genome-wide significance thresholds, by extrapolating from the length of chromosome 3 to the length of the whole genome. Chromosome 3 is approximately 198 million bp long and the whole genome, including X and Y is approximately 3.096 billion bp, or 15.6 times longer than chromosome 3 alone. Table 1 shows predicted genome-wide values for *m_e_* and significance thresholds based on this extrapolation for window-based tests alone, and Table 2 shows similar calculations for the combined analytic strategy including both single-SNP tests of common variants and window-based tests of rare variants. (Supplementary Table S1 shows the effective number of independent tests predicted for all of chromosome 3, and for the whole genome, corresponding to Fig.2). Because there is little correlation between rare genetic variants and common variants, the effective number of independent tests in Table 2 tends to be close to the sum of the number of independent window-based tests and the number of independent common-SNP tests.

**Table 1:** Estimated genome-wide significance thresholds for window-based tests of rare genetic variation, derived from simulations

MAF threshold defining rare variants	Test statistic	Number of windows on chromosome 3	Genome-wide estimate of the number of independent tests	Predicted genome-wide significance threshold
0.005	SKAT	71,179	682,646	7.32 × 10^−8^
0.005	Burden		1,127,774	4.43 × 10^−8^
0.005	SKAT-O		1,868,633	2.68 × 10^−8^
0.01	SKAT	74,156	615,665	8.12 × 10^−8^
0.01	Burden		1,319,873	3.79 × 10^−8^
0.01	SKAT-O		1,866,388	2.68 × 10^−8^
0.05	SKAT	81,858	741,391	6.74 × 10^−8^
0.05	Burden		1,062,330	4.71 × 10^−8^
0.05	SKAT-O		1,923,726	2.60 × 10^−8^

**Table 2:** Estimated genome-wide significance thresholds for a combined analytic strategy including window-based tests of rare genetic variation and single-marker tests for common variants

MAF threshold defining rare variants	Test statistic	Number of tests (single marker and windows) on chromosome 3	Genome-wide estimate of the number of independent tests	Predicted genome-wide significance threshold
0.005	SKAT	869,354	5,933,687	8.43 × 10^−9^
0.005	Burden		7,237,279	6.91 × 10^−9^
0.005	SKAT-O		8,547,380	5.85 × 10^−9^
0.01	SKAT	797,907	4,412,096	1.13 × 10^−8^
0.01	Burden		5,735,011	8.72 × 10^−9^
0.01	SKAT-O		6,019,458	8.31 × 10^−9^
0.05	SKAT	613,066	2,746,888	1.82 × 10^−8^
0.05	Burden		3,142,544	1.59 × 10^−8^
0.05	SKAT-O		4,306,272	1.16 × 10^−8^

Results are derived from simulations.

We have not provided standard errors of these estimates, because each point used for calculation of the regression parameters is based on subdivision of the same chromosome 3 data in different ways. We cannot therefore argue that the points are independent. However, the intervals (±1.96* standard error) shown in Figure3 are each calculated from disjoint sections of the chromosome.

For window-based tests, the largest influencing factor is the chosen test statistic. The SKAT test does not require as small a significance threshold as the other two methods. In contrast, for the combined analytic strategy of Table 2, the primary driver of significance threshold is the MAF. For MAF thresholds of 1% or larger, then significance threshold of 1 × 10^−8^ would be a reasonable choice for our data.

## Discussion

We have presented an empirical approach for estimating genome-wide significance thresholds for the analysis of sequencing data and rare genetic variation. Here, we have explored the impact of the choice of MAF threshold and the choice of test statistic on the necessary thresholds for window-based tests, and have shown that these factors have different patterns of dependence and will impact necessary genome-wide thresholds.

Therefore, for a chosen analytic strategy, the necessary threshold is likely to depend on the analysis plan, as well as on the design parameters such as ethnicity, sample size, or sequencing depth (because increased depth could identify more rare variants). If region-based tests are repeated under a large series of different models and assumptions, alteration of the significance thresholds would be necessary. In contrast, we believe that effects of sample size and depth on genome-wide significance thresholds will be small. Supplementary Figures S2 and S3 show that in our data, altering the sample size made no visible difference to the estimated significance thresholds. If sequence data are first imputed against a large population reference panel [Wood et al., 2013; Zheng et al., 2012], then additional read depth should lead to the addition of only a few extremely rare or singleton genetic variants. Nevertheless, it may be of interest to revisit the impact of sample size and depth in larger datasets.

However, the magnitude of effect of ethnic origin is currently unclear. Our data are based on UK individuals of European descent. Sequence variant correlation structure is dramatically different in more heterogeneous populations, for example, from sub-Saharan Africa. Reduced levels of linkage disequilibrium translate into higher numbers of uncorrelated variants, more independent tests and, hence a lower genome-wide significance threshold, and this would definitely be of interest to examine.

The same technique can be used to estimate genome-wide significance thresholds for single-marker tests of association for common SNPs, thereby enabling a comparison with previous estimates of genome-wide significance thresholds. These pilot data from the UK10K study identified 531,208 SNPs with MAF ≥ 0.05 on chromosome 3, in contrast to 464,048 in Europeans in the 1000 Genomes project (http://www.1000genomes.org, Supplementary Table S2). For MAF ≥ 0.005, the number of variants was very similar in the two datasets. Supplementary Figure S4 and Table S2 show that our estimated significance threshold for single-SNP tests with MAF ≥ 0.05 is 2.3 × 10^−8^. This is a little smaller than previous estimates for genotyping arrays (e.g., 7.2 × 10^−8^: [Dudbridge and Gusnanto, 2008]; 5 × 10^−8^: [Pe'er et al., 2008]), but in close agreement with a recent estimate based on 1,000 genomes sequencing data (3 × 10^−8^: [Li et al., 2012]).

Our calculations used portions of the genome of varying size to calculate the relationship between the number of tests performed and the effective number of independent tests. If warranted, it is therefore computationally tractable to repeat these calculations, for example, for studies with a significantly different study design than the one investigated here. In fact, we recommend that any study should consider undertaking simulations on a single chromosome to evaluate the necessary thresholds for their particular study design, especially if several window-based tests are anticipated.

A limitation with our extrapolation strategy is that the points used to calculate the regression line are not completely independent. Because we have repeatedly divided chromosome 3 into sections of different size, the same data have been used multiple times to obtain the significance thresholds for each distinct section size. Hence, we have not presented confidence intervals for our predictions; the mean values of the predictions should not be affected by dependence between the points.

We found good agreement between *m_e_* estimates from our simulations and two methods based on eigenvalues of the correlation matrix. There are, however, computational limitations associated with calculating eigenvalues in large matrices that limit the size of the genomic sections that can be explored with this approach. As an alternative strategy, there are at least three methods that calculate *m_e_* directly from the correlations without the need to calculate eigenvalues [Chen and Liu, 2011; Cheverud, 2001; Moskvina and Schmidt, 2008]. However, we found (data not shown) that these estimates did not agree well with our simulations, and hence these methods would have given biased estimates of significance thresholds.

Our correlation-based approach has used expected correlations between test statistics under the null hypothesis. In contrast, previous publications on this topic have focused on single-SNP tests and based their development on the correlation matrix between genotypes [Chen and Liu, 2011; Cheverud, 2001; Li and Ji, 2005; Moskvina and Schmidt, 2008] or the correlations between *P*-values [Li et al., 2012]; in the latter, the authors found that a sixth-order polynomial described the relationship between the *P*-value and the linkage disequilibrium. For window-based tests, correlations between genotypes are not the right metric, and correlations between *P*-values cannot be obtained without simulations, and hence we worked with the test-statistic correlations. Therefore, an explicit consideration of the relationship between test statistic correlations and genotype correlations, and the impact of the asymptotic distribution on estimators of *m_e_* could be a fruitful avenue for future research.

Assuming independence of genes, a genome-wide Bonferroni correction for gene-based burden tests in WES studies is 0.05/20,000 = 2.5 × 10^−6^. However, this threshold does not correct for single-variant tests performed on common-frequency sites or for possible correlation between genes. Our simulation-based approach would also be applicable to WES data, assuming that the genomic region used in the simulations has an average gene density.

In the new era of next-generation association studies, defining robust thresholds to declare significance is of paramount importance to help guard against false-positive signals. For window-based testing, the analysis choices will impact the necessary significance thresholds. The estimates we have obtained for genome-wide significance thresholds may vary if we had used another chromosome for our simulations, or if we had incorporated multiple window tests into our analytic strategy. However, for a combined strategy including single-marker tests of common variants combined with window-based tests for rare variants, we found that single-variant results are the principle drivers of significance thresholds and hence that significance thresholds depend little on the analytic choices for the windows and more on the MAF thresholds chosen.

We recommend that each study perform simulations to investigate the impact of their own analytic strategy on significance levels, and we suggest that sufficiently accurate predictions can be obtained by performing the simulations on a small portion of the whole genome and extrapolating to the full genome, thereby making such investigations feasible. An R package, QW signif, is available at http://cran.r-project.org to facilitate such calculations. We add the well-known caveat that all association signals require validation in independent data. Nevertheless, in European populations, a reasonable choice for WGS analyses that investigate association of complex traits with individual variants and locus-based aggregation of rare variants (using only one test statistic) can be expected to be between 0.6 × 10^−8^ and 1.5 × 10^−8^.

## Supporting Information

Disclaimer: Supplementary materials have been peer-reviewed but not copyedited.

Figures S1a – S1c. Correlations between 2000 adjacent window-based tests statistics for SKAT and burden tests and for two different MAF thresholds. Figure S2. Estimates of significance thresholds for window-based tests of rare variants, for different sample sizes. Results are derived using a MAF threshold of 0.01. Figure S3. Estimates of significance thresholds for window-based tests of rare variants, for different sample sizes. Results are derived using a MAF threshold of 0.05. Figure S4. Estimates of significance thresholds for single-SNP tests of common variants. Table S1. Estimated genome-wide significance thresholds for window-based testing, corresponding to results in Figure 2. Table S2. Estimated genome-wide significance thresholds for tests of single common SNPs.
