# Supplementary material for: Estimating Genome-Wide Significance for Whole-Genome Sequencing Studies
Source: Genet Epidemiol. 2014 Feb 14;38(4):281–90. doi: 10.1002/gepi.21797 (PMC4489336; doi:10.1002/gepi.21797)
Supplement: Supplementary file 1 [file gepi0038-0281-sd1.pdf]

Estimating genome-wide significance for whole genome sequencing studies

ChangJiang Xu, Ioanna Tachmazidou, Klaudia Walter, Antonio Ciampi, Eleftheria Zeggini, Celia M.T. Greenwood, and the UK10K Consortium

Supplemental Figures and Tables.

Pages 2-4: Supplemental Figures S1a – S1c.
Correlations between 2000 adjacent window-based tests statistics for SKAT and burden tests and for two different MAF thresholds.

Page 5. Supplementary Figure S2.
Estimates of significance thresholds for window-based tests of rare variants, for different sample sizes. Results are derived using a MAF threshold of 0.01.

Page 6: Supplementary Figure S3.
Estimates of significance thresholds for window-based tests of rare variants, for different sample sizes. Results are derived using a MAF threshold of 0.05.

Page 7: Supplemental Figure S4.
Estimates of significance thresholds for single-SNP tests of common variants.

Page 8: Supplemental Table S1.
Estimated genome-wide significance thresholds for window-based testing, corresponding to results in Figure 2.

Page 9: Supplemental Table S2.
Estimated genome-wide significance thresholds for tests of single common SNPs.

Supplemental Figures S1a – S1c.

Correlations between 2000 adjacent window-based tests statistics for SKAT and burden tests and for two different MAF thresholds. Since windows were defined to contain the same number of rare variants, the boundaries of the windows vary with MAF. Regions have been aligned so that the genomic region captured in the bottom row is contained within the genomic region at the top. The axes are the window numbers, counted from the 5' end of chromosome 3. Left column: SKAT tests. Right column: burden tests. Top row: MAF threshold =0.01; bottom row: MAF threshold=0.05. Gray: correlation>0.1; Yellow: correlation>0.35; Blue: correlation>0.5; Red: correlation>0.75. S1a: Windows 1-2000. S1b: Windows 36,250-38,250. S1c: Windows 54,500-56,500.

Figure S1a

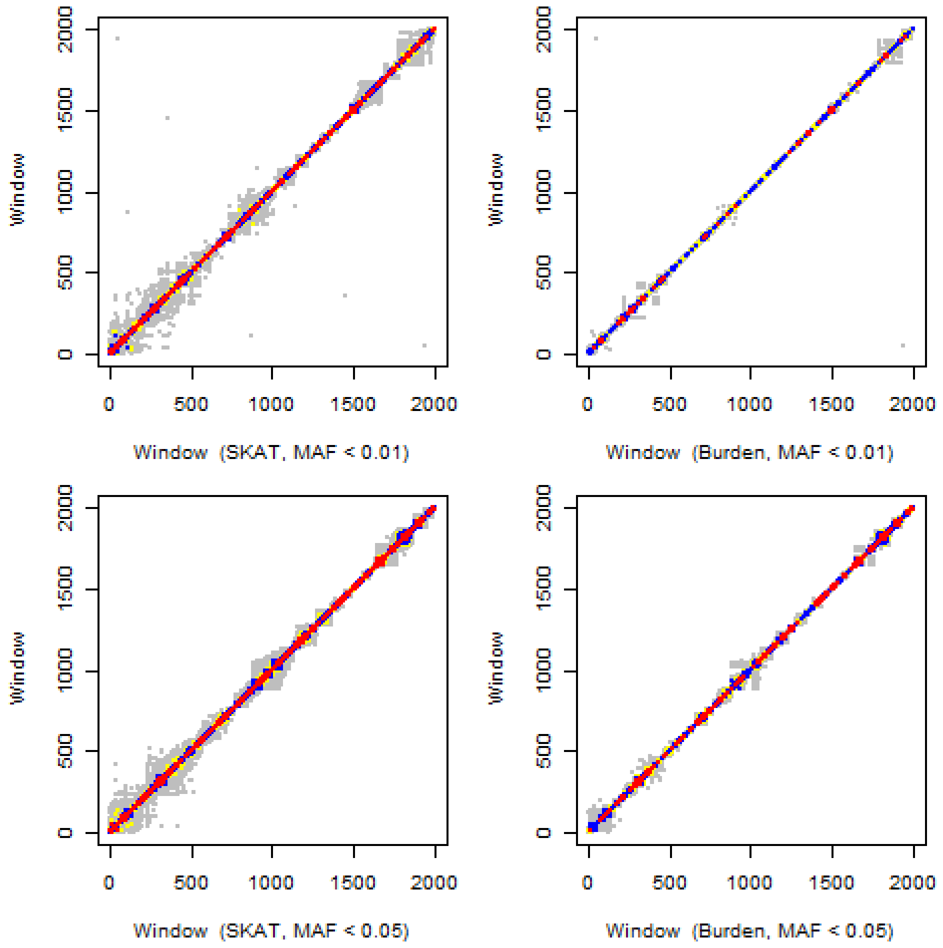

Figure S1b

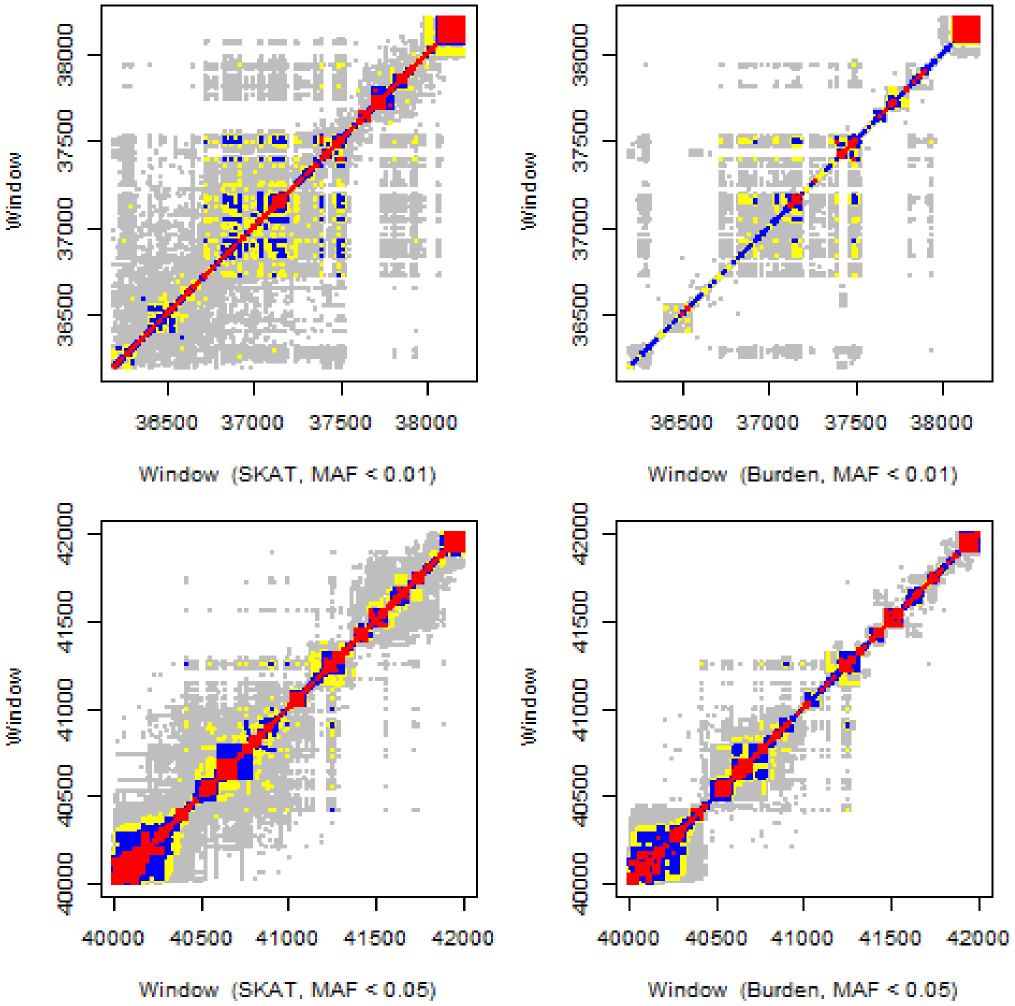
Window
38000
37500
37000
36500
36500 37000 37500 38000
Window (SKAT, MAF < 0.01)
Window (Burden, MAF < 0.01)
42000
41500
41000
40500
40000
40000 40500 41000 41500 42000
Window (SKAT, MAF < 0.05)
Window (Burden, MAF < 0.05)

Figure S1c

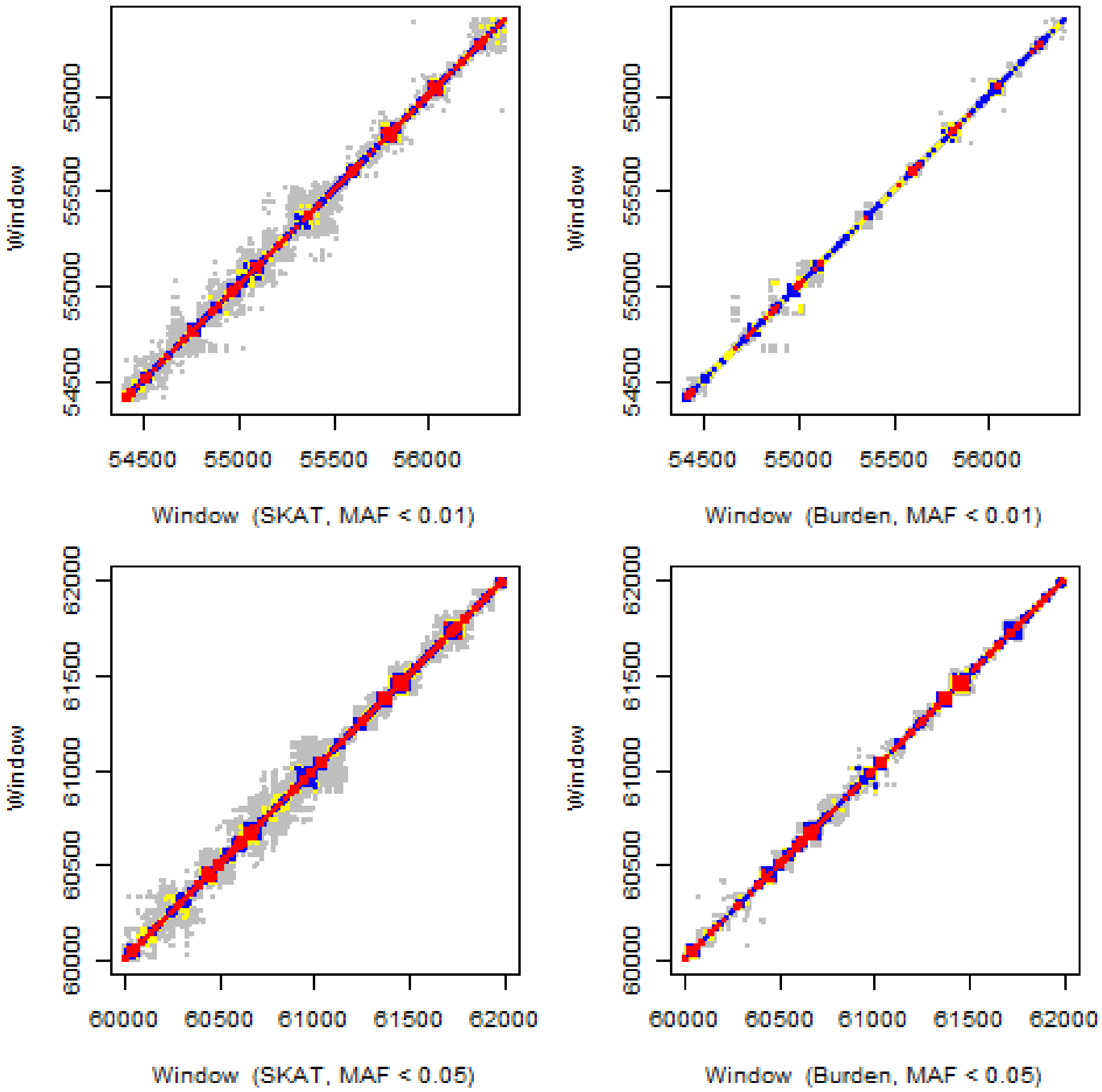
Window
54500
55000
55500
56000
Window (SKAT, MAF < 0.01)
Window (Burden, MAF < 0.01)
60000
60500
61000
61500
62000
Window (SKAT, MAF < 0.05)
Window (Burden, MAF < 0.05)

Supplementary Figure S2.

Estimates of significance thresholds for window-based tests of rare variants, for different sample sizes. Results are derived using a MAF threshold of 0.01. The x-axis is $-\log_{10}$ of the Bonferroni correction, i.e. $-\log_{10}(0.05/m)$, where m is the number of tests performed in sub-sections of chromosome 3 of varying sizes. The vertical axis shows the estimates of the significance thresholds obtained by each method or for each sample size. The mean, and 95% confidence interval for the mean, summarize the results across sections of chromosome 3 of the same size. Lines for different sample sizes lie almost on top of each other.

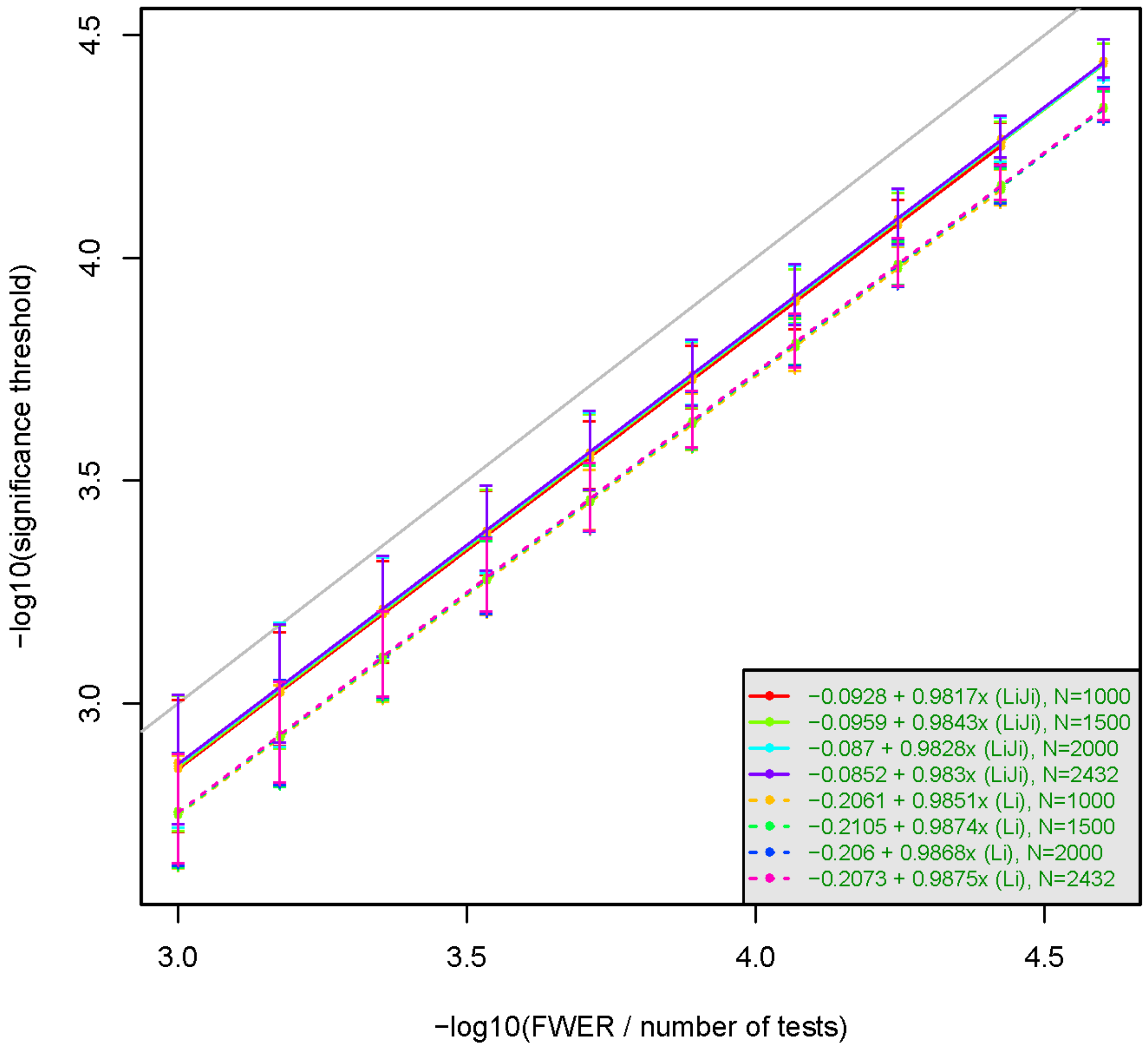

Supplementary Figure S3.
Estimates of significance thresholds for window-based tests of rare variants, for different sample sizes. Results are derived using a MAF threshold of 0.05. The x-axis is $-\log_{10}$ of the Bonferroni correction, i.e. $-\log_{10}(0.05/m)$, where m is the number of tests performed in sub-sections of chromosome 3 of varying sizes. The vertical axis shows the estimates of the significance thresholds obtained by each method or for each sample size. The mean, and 95% confidence interval for the mean, summarize the results across sections of chromosome 3 of the same size. Lines for different sample sizes lie almost on top of each other.

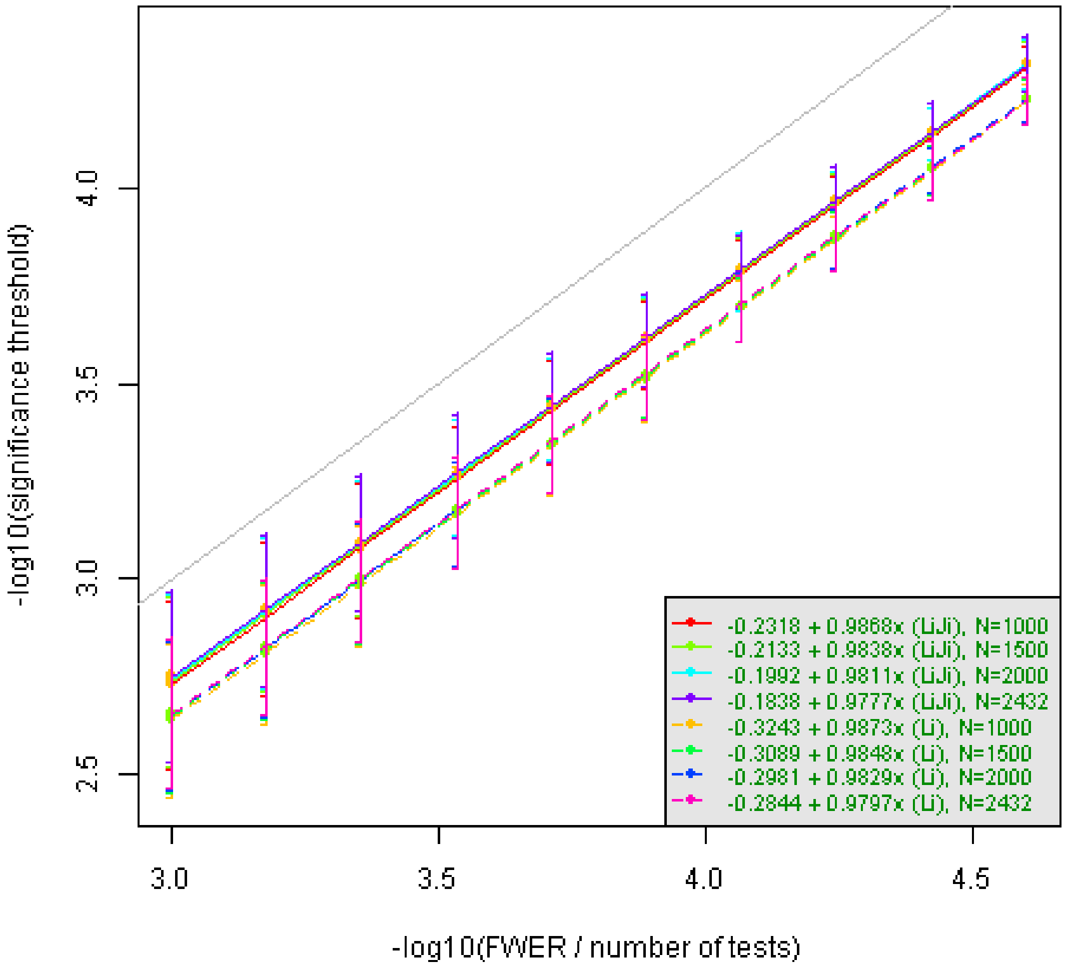

Supplemental Figure S4.
Estimates of significance thresholds for single-SNP tests of common variants. Results are derived using a sample size of 2,432. The x-axis is $-\log_{10}$ of the Bonferroni correction, i.e. $-\log_{10}(0.05/m)$, where m is the number of tests performed in sub-sections of chromosome 3 of varying sizes. The vertical axis shows the estimates of the significance thresholds obtained for three different MAF thresholds defining common variants. The mean, and 95% confidence interval for the mean, summarize the results across sections of chromosome 3 of the same size. Extrapolations to the approximate size of the full genome are shown.

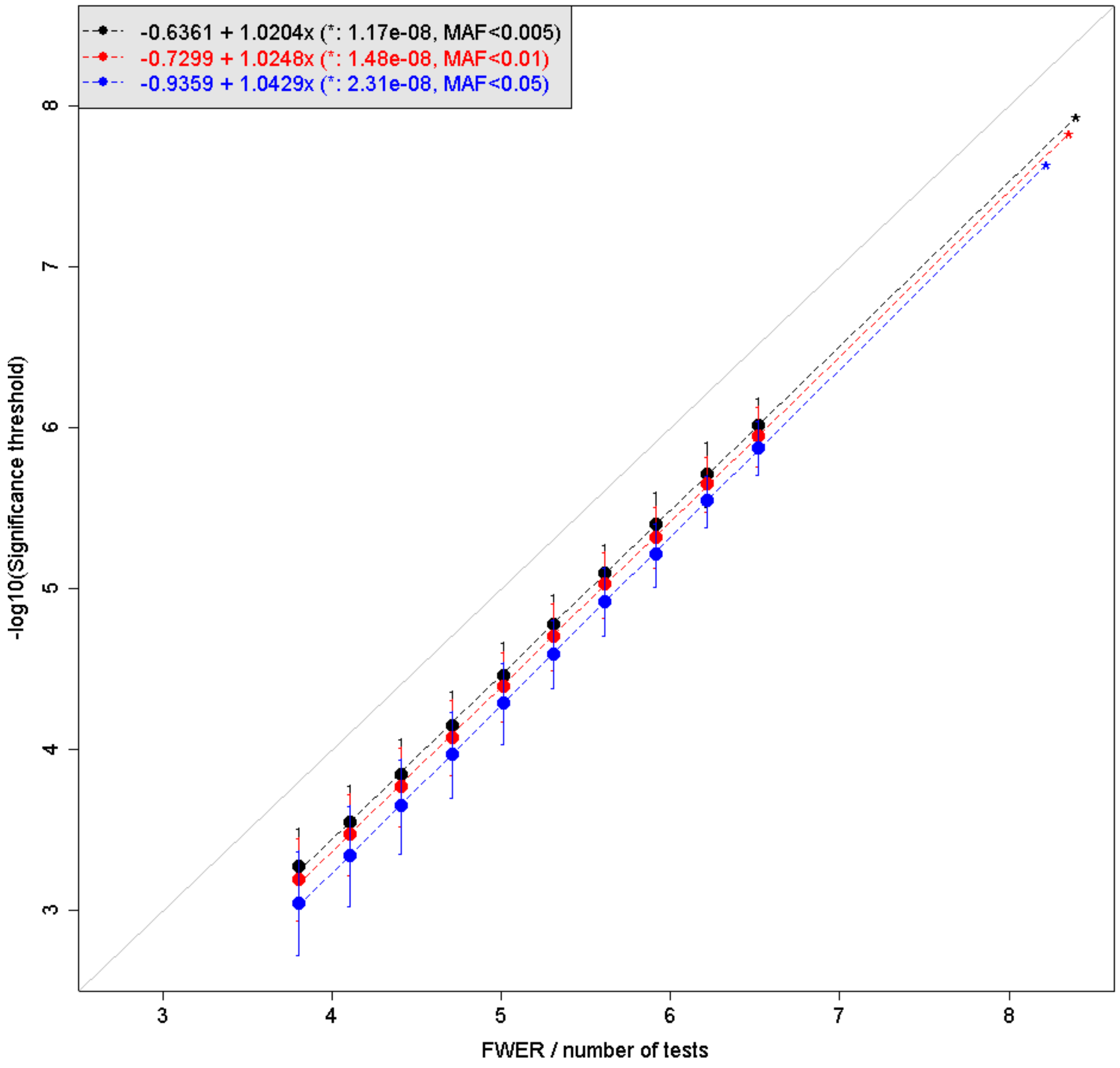

Supplemental Table S1.
Estimated genome-wide significance thresholds for window-based testing, corresponding to results in Figure 2. Estimates are derived from expected correlations.

MAF threshold defining rare variants	Test statistic	Method for estimating effective number of independent tests	Estimate of the number of independent tests on chromosome 3	Genome-wide estimate of the number of independent tests	Predicted genome-wide significance threshold
0.01	SKAT	Li and Ji	48,606	730,252	6.85e-08
0.01	SKAT	Li et al.	38,907	590,030	8.47e-08
0.01	Burden	Li and Ji	66,577	1,035,006	4.83e-08
0.01	Burden	Li et al.	54,664	850,773	5.88e-08
0.05	SKAT	Li and Ji	40,332	602,977	8.29e-08
0.05	SKAT	Li et al.	32,872	493,618	10.13e-08
0.05	Burden	Li and Ji	58,284	903,229	5.54e-08
0.05	Burden	Li et al.	48,322	750,843	6.66e-08

Supplemental Table S2.
Estimated genome-wide significance thresholds for tests of single common SNPs. Results correspond to Supplemental Figure S4. Results are obtained from simulations under the null hypothesis on chromosome 3. For comparison, the last row shows the number of common SNPs in 1000 Genomes European data.

**MAF threshold defining common SNPs**	**MAF≥0.005**	**MAF≥0.01**	**MAF≥0.05**
Number of common SNPs on chromosome 3 in UK10K pilot data	798,175	723,751	531,208
Estimated number of common SNPs genome-wide	12,451,530	11,290,516	8,286,845
Estimated number of independent test statistics	4,268,111	3,385,954	2,164,077
Estimated significance threshold	1.17e-08	1.48e-08	2.31e-08
Number of common SNPs on chromosome 3 from 1000 Genomes European data	773,690	665,639	464,048
